# Wenxin Keli regulates energy metabolism and improves Cx43 via the AMPK/SIRT1/PGC-1α pathway

**DOI:** 10.3389/fphar.2025.1624595

**Published:** 2025-09-09

**Authors:** Xiyuan Cui, Lixia Lou, Bo Nie, Yizhou Zhao, Jiuli Zhao, Ding Yang, Zhe Wang, Shaoqing Zheng, Aiming Wu

**Affiliations:** Dongzhimen Hospital Beijing University of Chinese Medicine/Key Laboratory of Chinese Internal Medicine of Ministry of Education and Beijing, Beijing, China

**Keywords:** PGC-1α, SIRT1, Wenxin Keli, heart failure, energy metabolism, connexin 43

## Abstract

**Background:**

Research has underscored the significance of targeting energy metabolic remodeling in heart failure (HF) as a crucial therapeutic avenue in recent years. Following the onset of heart failure, dysregulated energy metabolism induces multiple adverse effects, exemplified by the reduced expression of connexin 43 (Cx43)—a gap junction protein requiring substantial ATP for phosphorylation modification—in rats with post-myocardial infarction (MI) heart failure. In this study, we report that Wenxin Keli (also known as Wenxin granule), a clinically available Chinese patent medicine used for preventing and treating heart failure–related arrhythmias, modulates energy metabolism and improves Cx43 function by activating AMPK/SIRT1/PGC-1α signaling pathway. However, the pathological alterations after heart failure are intricate, and the underlying mechanism through which Wenxin Keli exerts its therapeutic effect on heart failure remains to be further elucidated.

**Methods:**

A post-myocardial infarction heart failure rat model was established via left anterior descending coronary artery ligation. Cardiac function was evaluated 4 weeks later using echocardiography, HE, and Masson trichrome staining. ELISA was employed to detect energy metabolism-related indices, while WB analysis was used to quantify the expression levels of proteins, including SIRT1, PGC-1α, and Cx43. IHC was further utilized to assess Cx43 protein content in tissue sections. Ventricular fibrillation (VF) was induced to determine the VF threshold, providing insights into arrhythmogenic susceptibility.

**Results:**

Wenxin Keli enhances energy metabolism and improves Cx43 function in post-MI heart failure rats by activating the AMPK/SIRT1/PGC-1α signaling pathway. Specifically, Wenxin Keli stimulates the SIRT1/PGC-1α axis, promoting interaction between PGC-1α and PPARs and ERRs. This dual mechanism addresses the combined impairments in fatty acid oxidation and glucose utilization after heart failure, restoring mitochondrial oxidative phosphorylation and increasing ATP production through the TCA cycle. Furthermore, Wenxin Keli boosts the positive regulatory effect of SIRT1 on PGC-1α by upregulating AMPK phosphorylation, thereby further activating the AMPK/SIRT1/PGC-1α signaling pathway and creating a positive feedback loop.

**Conclusion:**

Wenxin Keli exhibits multi-target regulation of energy metabolic disorders in post-myocardial infarction heart failure while protecting Cx43. Its core mechanism is activating the AMPK/SIRT1/PGC-1α signalling pathway and its downstream regulatory network.

## 1 Introduction

Heart failure represents the final outcome of cardiovascular diseases, affecting approximately 40 million people worldwide (Cannata et al., 2025; Baman and Ahmad, 2020). It has complex causes, with coronary artery obstruction being a major factor in many cases. The main pathophysiological change is ventricular remodelling (Frantz et al., 2022). Dysregulated energy metabolism is recognized as a key factor in the progression of heart failure. After heart failure begins, abnormal energy metabolism occurs, marked by a shift from fatty acid to glucose metabolism (Shehadeh et al., 2025). However, the reduction in fatty acid oxidation is not matched by an increase in glucose oxidation. The uncoupled oxidation process increases glycolysis, generating less energy. A healthy heart needs to convert chemical energy from fatty acid and glucose oxidation into the mechanical energy of myofibrils to sustain its pumping function. Insufficient energy supply weakens cardiac pumping, ultimately causing mechanical energy failure (Bertero and Maack, 2018). Additionally, inadequate energy can negatively affect communication between cardiomyocytes, significantly raising the risk of ventricular arrhythmia. This mechanism mainly involves impairment of Cx43 function caused by reduced ATP, an energy source, following heart failure. Cx43 is a transmembrane protein crucial for forming gap junctions between cardiomyocytes and is vital for intercellular communication (Kléber and Jin, 2021; Liu et al., 2022). The decoupling of Cx43 can lead to abnormal intercellular connections and arrhythmias. Moreover, Cx43 coupling requires phosphorylation, which demands a substantial amount of ATP as a phosphate donor (Sato et al., 2023; Boengler et al., 2012). From this perspective, energy is of utmost importance for the heart.

In the molecular mechanism of myocardial cell energy metabolism, peroxisome proliferator-activated receptor γ coactivator-1 (PGC-1α) is recognised as a critical mediator of mitochondrial energy metabolism (Vannuchi and Pisani, 2025). The two most significant upstream target proteins that can activate and enhance the regulation of energy metabolism by PGC-1α are AMPK and SIRT1 (Palabiyik and Palabiyik, 2025). AMPK increases fatty acid metabolism and energy utilisation efficiency by phosphorylating and activating PGC-1α. Relevant literature indicates that the expression of PGC-1α is significantly reduced following heart failure (Vannuchi and Pisani, 2025). Since PGC-1α interacts with various coactivator nuclear receptors, such as PPARs and ERRs, to regulate mitochondrial oxidative metabolism (Vannuchi and Pisani, 2025; Fan and Evans, 2015), the reduced interaction between PGC-1α and PPARs and ERRs after heart failure negatively affects fatty acid oxidation and glucose uptake (Vannuchi and Pisani, 2025; Cantó and Auwerx, 2009). Therefore, investigating the molecular mechanisms of heart failure via the AMPK/SIRT1/PGC-1α energy metabolism pathway is a critically important research direction.

Wenxin Keli (also known as Wenxin granule) is the first Chinese herbal medicine formulation approved for antiarrhythmic indications by the China Food and Drug Administration (CFDA). Systematic reviews and randomised controlled trials (RCTs) prove that Wenxin Keli is a safe and effective treatment for patients with ventricular premature beats without severe structural heart disease. (Jiang et al., 2022; He et al., 2016). Research has convincingly demonstrated the clinical efficacy of Wenxin Keli, prompting detailed investigation into its high-performance liquid chromatography (HPLC) analysis and the mechanisms behind its effects. The literature identifies the main pharmacologically active components entering the bloodstream from Wenxin Keli as notoginsenoside R1, ginsenoside Rg1, and ginsenoside Rb1. (Liang et al., 2024; Mi et al., 2022; Li et al., 2021). Regarding mechanistic insights, Wenxin Keli effectively inhibits peak sodium current (I_n_a, peak), late sodium current (I_n_a, late), and L-type calcium current (I_n_a, L). It modulates transient outward potassium current (I_to_) and regulates hyperpolarisation-activated cyclic nucleotide-gated (HCN) channels. (Hu et al., 2016; Hou et al., 2016; Minoura et al., 2013; Xue et al., 2013). Additionally, Wenxin Keli protects against vascular endothelial cell damage, alleviates myocardial ischaemia, and prevents myocardial fibrosis and hypertrophy. It does so through multiple mechanisms: suppressing inflammatory responses, reducing oxidative stress, regulating vascular vasomotor function, and attenuating cardiomyocyte apoptosis. Collectively, these actions maintain normal cardiac structural integrity and functional homeostasis. (Tian et al., 2018). However, the pharmacological mechanisms underlying its effects on energy metabolism remain unclear. This study aims to elucidate the precise molecular mechanisms by which Wenxin Keli regulates myocardial energy metabolism and preserves Cx43 function, mediated by the AMPK/SIRT1/PGC-1α signalling axis.

## 2 Materials and methods

### 2.1 Animal experiment

Sixty male SPF-grade rats with body weights ranging from (210 ± 20) g were procured from Beijing Vital River Laboratory Animal Technology Co., Ltd. The license number is SCXK (Beijing) 2021–0006. The animal study was approved by the Ethics Committee of Dongzhimen Hospital, Beijing University of Chinese Medicine (Approval No. 23-11). All procedures complied with the NIH Guide for the Care and Use of Laboratory Animals.

A left coronary artery ligation model was established in rats to induce post- MI heart failure (Riehle and Bauersachs, 2019; De Villiers and Riley, 2020). Relevant indicators were assessed 4 weeks after surgery. Initially, the rats were anaesthetised with 1% pentobarbital sodium (50 mg/kg, intraperitoneal injection). They were then positioned supine and stabilised. Tracheal intubation was performed, followed by a ventilator set to an inspiration-expiration ratio of 1: 2, delivering 7 mL of gas per minute at a respiration rate of 80 breaths per minute to maintain vital signs. Povidone-iodine was used for disinfection after moistening the hair in the precordial area and shaving the skin. A transverse thoracotomy was performed at the third and fourth intercostal spaces along the left border of the sternum in the precordial region. The pleura was incised, the thymus removed, and a chest expander used to expose the surgical field. Ligation was performed 2 mm below the bifurcation of the left auricle artery; the ligated myocardial tissue turned white, indicating ischaemia and successful myocardial infarction model establishment. The chest cavity was then closed and sutured layer by layer. The entire surgical procedure strictly adhered to aseptic principles. A sham operation was conducted as the control group, where only the thread was passed through without ligation. Immediately after the operation, 0.2 mL of lignocaine and 40 U of penicillin sodium were administered intraperitoneally for three consecutive days to reduce the risk of infection. Pathological Q waves were observed on the electrocardiogram 24 h post-operation. The presence of pathological Q waves in leads V3-V6, I, AVL, and either V1 or V2 on the electrocardiogram served as the criteria for model qualification and group inclusion. Rats were randomly assigned to three groups: the sham group, the model group, and the drug-intervention groups, which included low-dose (Wenxin Keli-L), high-dose Wenxin Keli, and a group receiving trimetazidine, with each group containing 12 rats. The dosing regimens were as follows: the low-dose Wenxin Keli group, 2.7 g/kg for the high-dose Wenxin Keli group, and 10 mg/kg for the trimetazidine group. Intragastric administration began 24 h post-operation and continued daily for 4 weeks according to the assigned group.

### 2.2 Echocardiography

After 4 weeks of treatment, two-dimensional M-mode ventricular images of the long and short axes of the left ventricle were obtained using an ultrasonic imaging system positioned at the left margin of the sternum. The thicknesses of the anterior and posterior walls, the internal diameter of the left ventricle, and the volumes of the left ventricle during systole and diastole were measured. Subsequently, the relevant cardiac function parameters were calculated. Three cardiac cycles were selected for each animal, and the average values were computed.

### 2.3 HE and masson

The prepared myocardial tissue sections were placed on a slide warmer at 60 °C for 1 hour to facilitate dewaxing. They were successively immersed in xylene I, II, and III for 15 min each for thorough dewaxing. After that, the sections were hydrated by ethanol gradients with different concentrations for 2 min each, rinsed with tap water for 5 min, and stained as per the reagent instructions. For the Masson staining, the procedures for baking the slides, dewaxing, and gradient ethanol hydration were identical to those used for HE staining, with subsequent steps performed as per the guidelines. After staining, an optical microscope was employed to observe the myocardial infarction, and the collagen volume fraction (CVF) was calculated using the formula: CVF = collagen area/(myocardial tissue area + collagen area).

### 2.4 Ventricular fibrillation threshold

Rats were anesthetized via intraperitoneal injection, intubated tracheally, and subsequently connected to a ventilator and a BL-420 biological function system. After selecting the system software, the user should navigate to the menu option “input signal/channel/electrocardiogram” to display the electrocardiogram waveform. The chest was incised along the fourth and fifth intercostal spaces to expose the heart. The positive and negative electrodes of the stimulating electrode were placed at the heart’s apex and about 3 mm towards the base, respectively. The electrode stimulation was set as coarse voltage, train stimulation with 10 waves, and a starting intensity of 1 V. After image stabilization, electrical stimulation began. The induction of ventricular fibrillation by electrical stimulation up to 15 V was recorded, and the voltage at the first induction was defined as the ventricular fibrillation threshold.

### 2.5 Elisa

To extract samples from the myocardial infarction border zone in rats, the tissue was first subjected to two washes with pre-cooled PBS solution while on ice. A suitable quantity of the tissue was then combined with a PBS solution that contained protease inhibitors, maintaining a ratio of 1 mL–100 μL. Next, magnetic beads were incorporated, and the resulting mixture was homogenised with the aid of a homogeniser. The homogenate was then centrifuged at 4 °C, and the supernatant was aliquoted and analyzed according to the instructions provided by the ELISA reagent. ATP kit. (Enzyme-linked Biotechnology Co., Ltd., lot number: YJ582034), ADP kit (Enzyme-linked Biotechnology Co., Ltd., lot number: YJ594033), AMP kit (Enzyme-linked Biotechnology Co., Ltd., lot number: YJ539432), mitochondrial respiratory chain complex I, II, IV, V kits (Beijing Dongge Boye Biotechnology Co., Ltd., lot numbers: DG21009D - 96T, DG21011D - 96T, DG21054D - 96T, DG21056D - 96T), Rat NT - proBNP ELISA Kit (Elabscience, E − EL - R3023–96T)

### 2.6 IHC

In the early stages of immunohistochemistry (IHC), the procedures for baking the slides, dewaxing, and gradient ethanol hydration are identical to those used in hematoxylin and eosin (HE) staining and Masson’s trichrome staining. Subsequently, the slides are rinsed with phosphate-buffered saline (PBS) and immersed in sodium citrate antigen retrieval solution for antigen retrieval. After another rinse, the primary antibody is added dropwise and incubated overnight. The following day, the corresponding secondary antibody is added, the slides are rinsed with PBS, colour development is performed using DAB, and finally, the slides are mounted.

### 2.7 Western blot

Rat myocardial tissue was homogenized to obtain supernatant, and protein concentration was adjusted to 5 μg/μL using BCA quantification. Prepare the SDS-PAGE gel using the SDS-PAGE kit (7.5%), and add 4 μL of protein sample to each well in equal amounts. Proteins were electrophoresed and transferred to a PVDF membrane, then blocked, incubated with primary antibody overnight at 4 °C, and secondary antibody for 1 h. After TBST washing, bands were visualised via ECL chemiluminescence and documented, with grey values analysed using ImageJ v1.8.0. CD36, PFK, PDK, GLUT4, PPARδ, ERRα, PDH, and p-AMPK were purchased from CST (Cell Signaling Technology), with the catalog numbers being (#28109, #13123, #3062, #2213, #74076, #13826, #3205, #2535) respectively; CPT-1α, PPARα, Cx43, SIRT1, and AMPK were purchased from abcam, with the catalog numbers being (ab234111, ab24509, ab11370, ab110304, ab32047) respectively; p-Cx43(S282) and PGC-1α were purchased from Affinity, with the catalog numbers being (AF8230, AF5395) respectively; ERRγ and ATP5D were purchased from Prointech, with the catalog numbers being (14017-1-AP, 14893-1-AP) respectively.

### 2.8 Data and statistical analysis

Statistical analysis used SPSS. Data were expressed as mean ± SD. Normality and homogeneity of variance were tested first. For normally distributed continuous variables, one-way ANOVA was applied: LSD post-hoc tests for homogeneous variance, Tamhane’s T2 for heterogeneous variance. Non-parametric tests were used for non-normally distributed variables. Spearman’s correlation analyzes relationships. A P-value <0.05 indicated statistical significance.

## 3 Result

### 3.1 Ultrasonic cardiac function assessment, cardiac histology, and N-terminal pro-brain natriuretic peptide (NT-proBNP) in rats of each group

**FIGURE 1:**
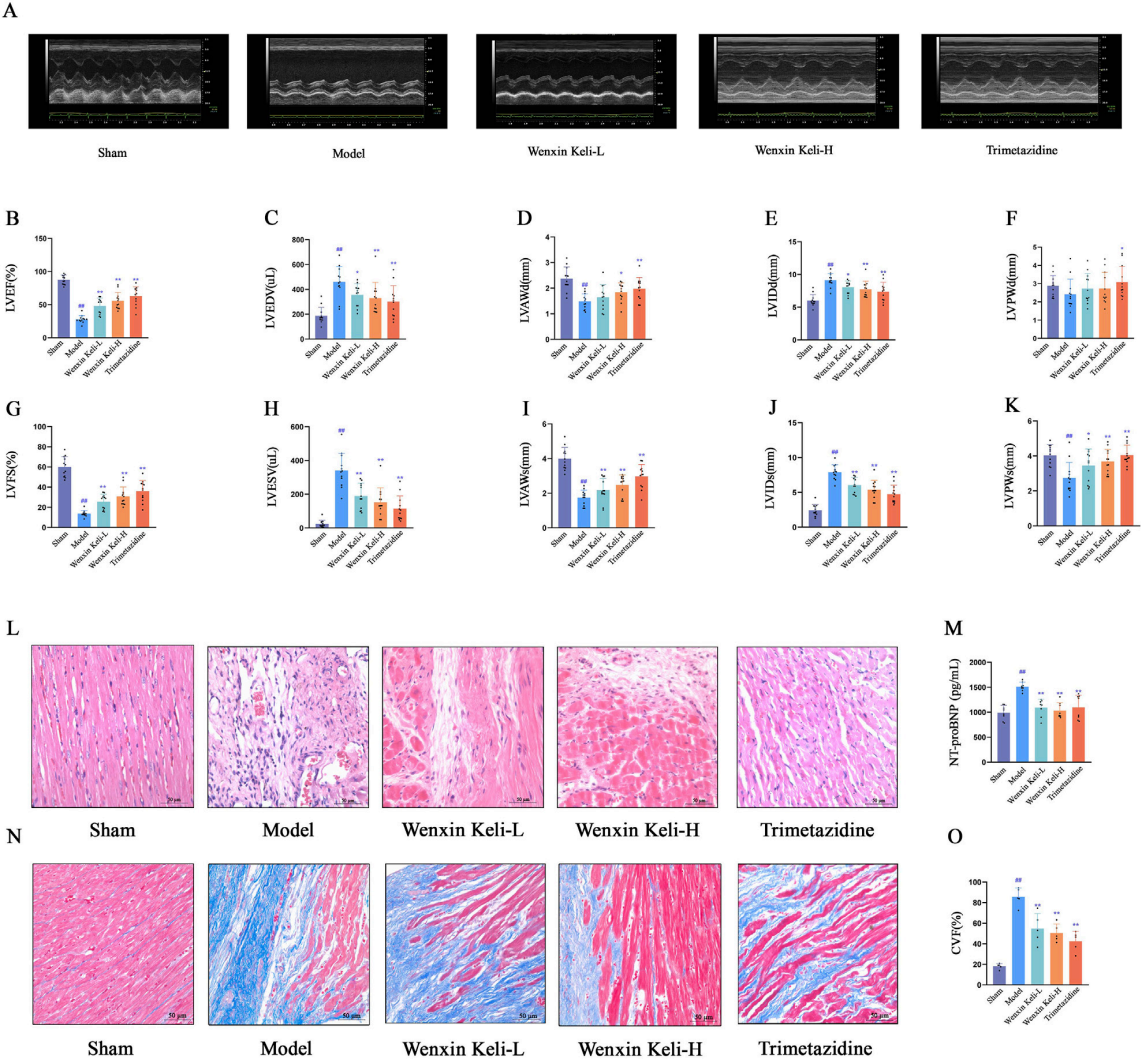
Effects of Wenxin Keli and trimetazidine on cardiac remodeling/dysfunction in myocardial infarction-induced heart failure rats **(A)** Echocardiography measured cardiac function, with representative images shown, **(B–K)** LVEF, LVFS, and other indices analyzed via echocardiography (n = 12), **(L**,**N)** HE and Masson staining results, **(M)** NT-proBNP detected by ELISA (n = 7), **(O)** Collagen fiber deposition quantified via ImageJ (n = 5). ^#^ *p* < 0.05, ^##^ *p* < 0.01 vs. Sham group; *p < 0.05, **p < 0.01 vs. Model group.

At 4 weeks post-operation, the model group had significantly lower LVEF and LVFS than the sham group, with structural changes including thinner ventricular walls and enlarged cardiac cavities (Figures 1A–K). Histological examination via HE staining revealed myocardial fiber fractures, widened intercostal spaces, nuclear pyknosis, and abnormal nuclear morphologies (Figure 1L). Additionally, NT-proBNP levels were elevated (Figure 1M). Masson staining demonstrated extensive collagen deposition, pronounced myocardial fibrosis, and an increased myocardial collagen volume fraction (Figures 1N,O). Notably, these pathological features were alleviated to varying degrees in the drug treatment group.

### 3.2 Detect the contents of ATP, AMP, and ADP, as well as the activities of mitochondrial respiratory chain complexes I, II, IV, and V that reflect the mitochondrial oxidative capacity, and the ATP synthase subunit ATP5D

**FIGURE 2:**
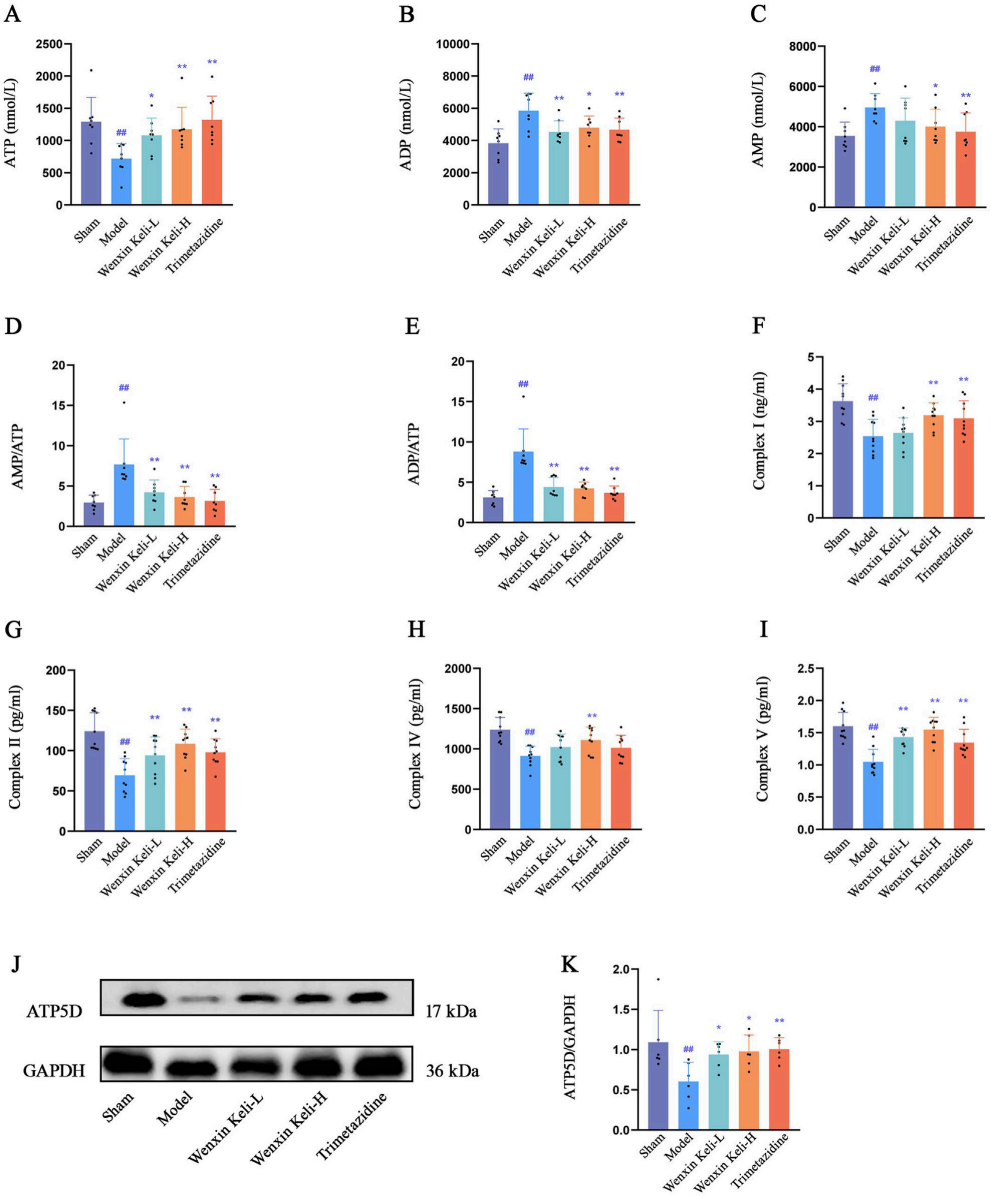
Effects of Wenxin Keli and trimetazidine on energy metabolism in myocardial infarction-induced heart failure rats **(A**–**E)** ELISA-detected ATP, AMP, ADP levels (n = 8), **(F**–**I)** mitochondrial complexes I, II, IV, V measured via ELISA (n = 10), **(J**,**K)** WB analyzed ATP5D expression, ImageJ-quantified (n = 6).

The relevant energy indicators were detected using the ELISA method, while the expression level of ATP5D was assessed via WB. Compared to sham-operated controls, the model group exhibited significantly reduced ATP levels and impaired activities of mitochondrial complexes I, II, IV, and V (Figures 2F–I). Conversely, the ratios of AMP, ADP, AMP/ATP, and ADP/ATP were notably increased (Figures 2A–E), and the expression of ATP5D was diminished (Figures 2J,K). After administering the drug groups, there was an increase in the levels of ATP as well as Complexes I, II, IV, and V. Conversely, the ratios of AMP, ADP, AMP/ATP, and ADP/ATP showed a decrease. These results indicate that drug intervention markedly boosts myocardial energy production in rats experiencing heart failure after a myocardial infarction.

### 3.3 AMPK, SIRT1, PGC-1α and Co-regulatory factors PPARs and ERRs

**FIGURE 3:**
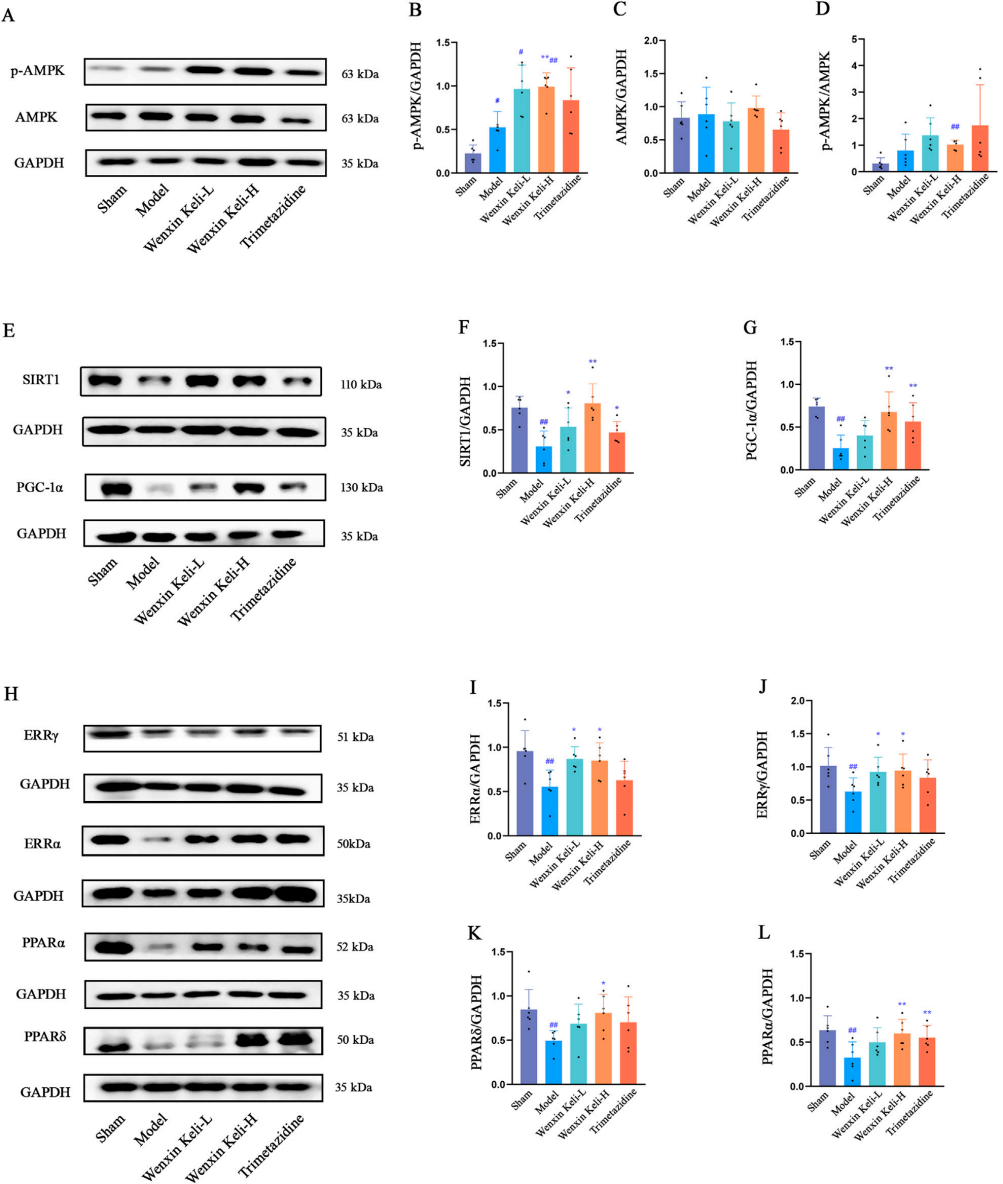
Effects of Wenxin Keli and trimetazidine on AMPK/SIRT1/PGC-1α pathway proteins in myocardial infarction-induced heart failure rats: **(A**–**G)** WB analyzed AMPK, SIRT1, PGC-1α expression, quantified via ImageJ (n = 6), **(H**–**L)** PPARs, ERRs detected similarly by WB with ImageJ (n = 6).

The AMPK/SIRT1/PGC-1α pathway is critical for energy metabolism regulation. WB analysis showed that the model group had reduced SIRT1 and PGC-1α levels, stable AMPK expression, and a slight increase in p-AMPK compared to the sham group. Wenxin Keli treatment elevated p-AMPK, with a notable increase in the p-AMPK/AMPK ratio in the high-dose group versus the sham group (Figures 3A–D). Additionally, drug intervention groups showed varying increases in SIRT1 and PGC-1α expression compared to the model group (Figures 3E–G). Wenxin Keli elevated p-AMPK levels, whereas trimetazidine caused no significant change. The high-dose Wenxin group exhibited a notable increase in the p-AMPK/AMPK ratio versus the sham group. For downstream targets, model rats had reduced PPARs/ERRs expression vs. sham controls, which were restored to varying degrees by Wenxin Keli (Figure 3H–L), highlighting its role in activating this energy regulatory pathway.

### 3.4 Expression of proteins regulating fatty acid transport and GLUT4 as well as related regulatory enzymes of OXPHOS

**FIGURE 4:**
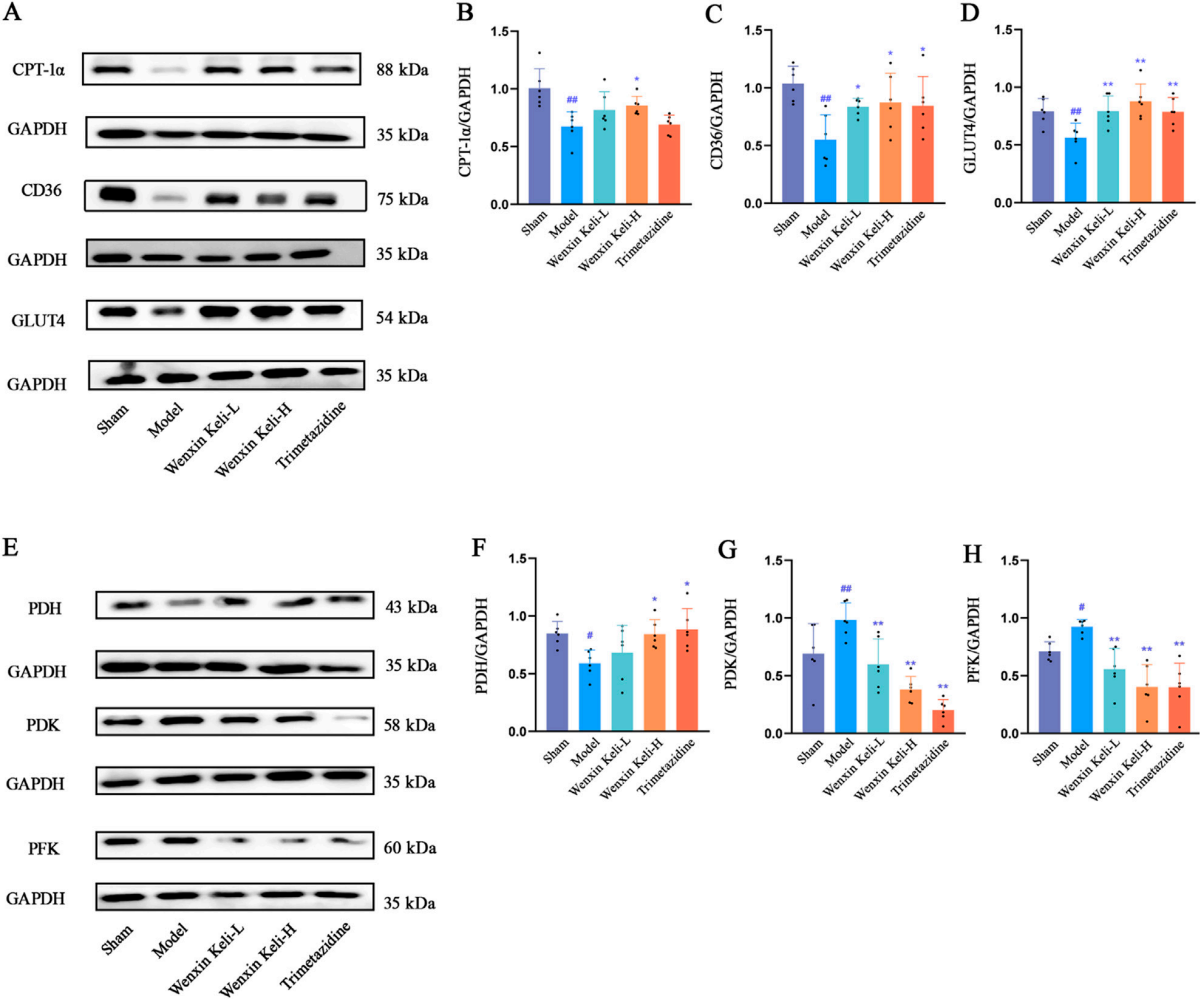
Effects of Wenxin Keli and trimetazidine on CD36, CPT-1α, GLUT4, PDH, PDK, and PFK proteins in myocardial infarction-induced heart failure rats, **(A**–**D)** WB analyzed CD36, CPT-1α, GLUT4 expression, quantified via ImageJ (n = 6), **(E**–**H)** PDH, PDK, PFK detected with ImageJ (n = 6).

WB analysis showed significantly lower CD36, CPT-1α, and GLUT4 levels in the model group versus the sham group. Wenxin Keli increased fatty acid transport proteins CD36/CPT-1α, while both Wenxin Keli and trimetazidine significantly upregulated GLUT4 expression (Figures 4A–D). Additionally, the expression levels of PDH, PDK, and PFK were evaluated by WB. Compared with the sham group, the model group had lower PDH expression, while PDK and PFK expressions were higher (Figures 4E–H). Following treatment with the drug groups, the trends in protein expression were reversed to varying degrees.

### 3.5 Ventricular fibrillation threshold

**FIGURE 5:**
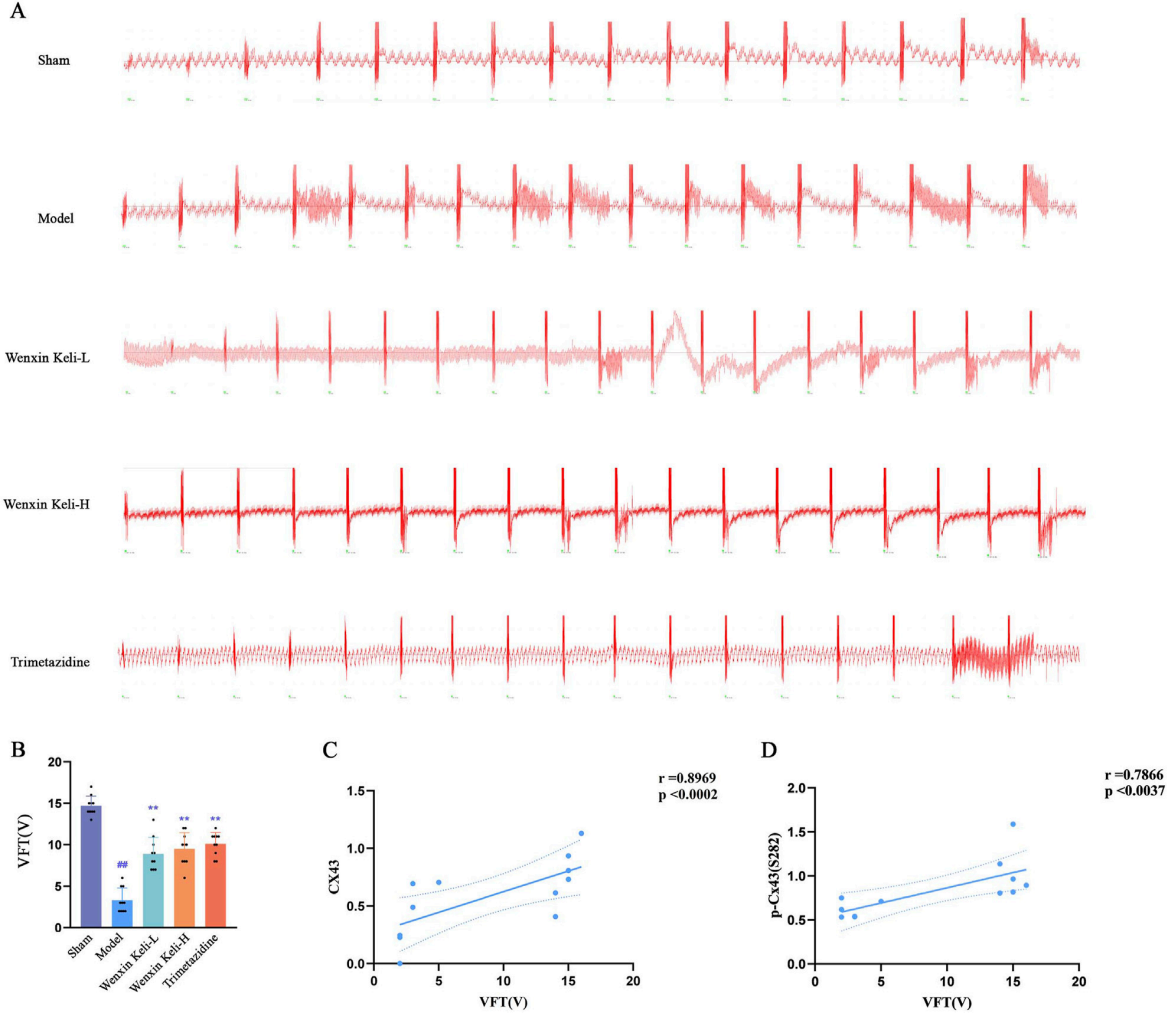
Effects of Wenxin Keli and trimetazidine on ventricular tachycardia duration in myocardial infarction-induced heart failure rats **(A)** Image of the Ventricular Fibrillation (VF) threshold, **(B)** VF threshold incidence calculated (n = 10), **(C,D)** correlation coefficients of Cx43, p-Cx43(S282) expressions.

The electrophysiological parameters, including the ventricular fibrillation threshold, arrhythmia induction rate, and duration, which reflect cardiac electrical coupling function, were assessed using a biological function experimental system. In this system, the coarse voltage mode was conducted with a frequency of 30 Hz, burst stimulation mode, pulse width of 5 m, an initial intensity of 1 V, and a primary cycle of 30 s (adjusted according to experimental requirements) (Figures 5A,B). Cx43 and p-Cx43(S282) expression levels showed strong positive correlations with ventricular fibrillation threshold (Figures 5C,D). Model rats had a lower ventricular fibrillation threshold than sham controls. At the same time, drug-treated groups showed significant increases, indicating Wenxin Keli and Trimetazidine improved electrical coupling in post-MI heart failure.

### 3.6 Detection of the expression of Cx43 and its correlation with energy metabolism indicators

**FIGURE 6:**
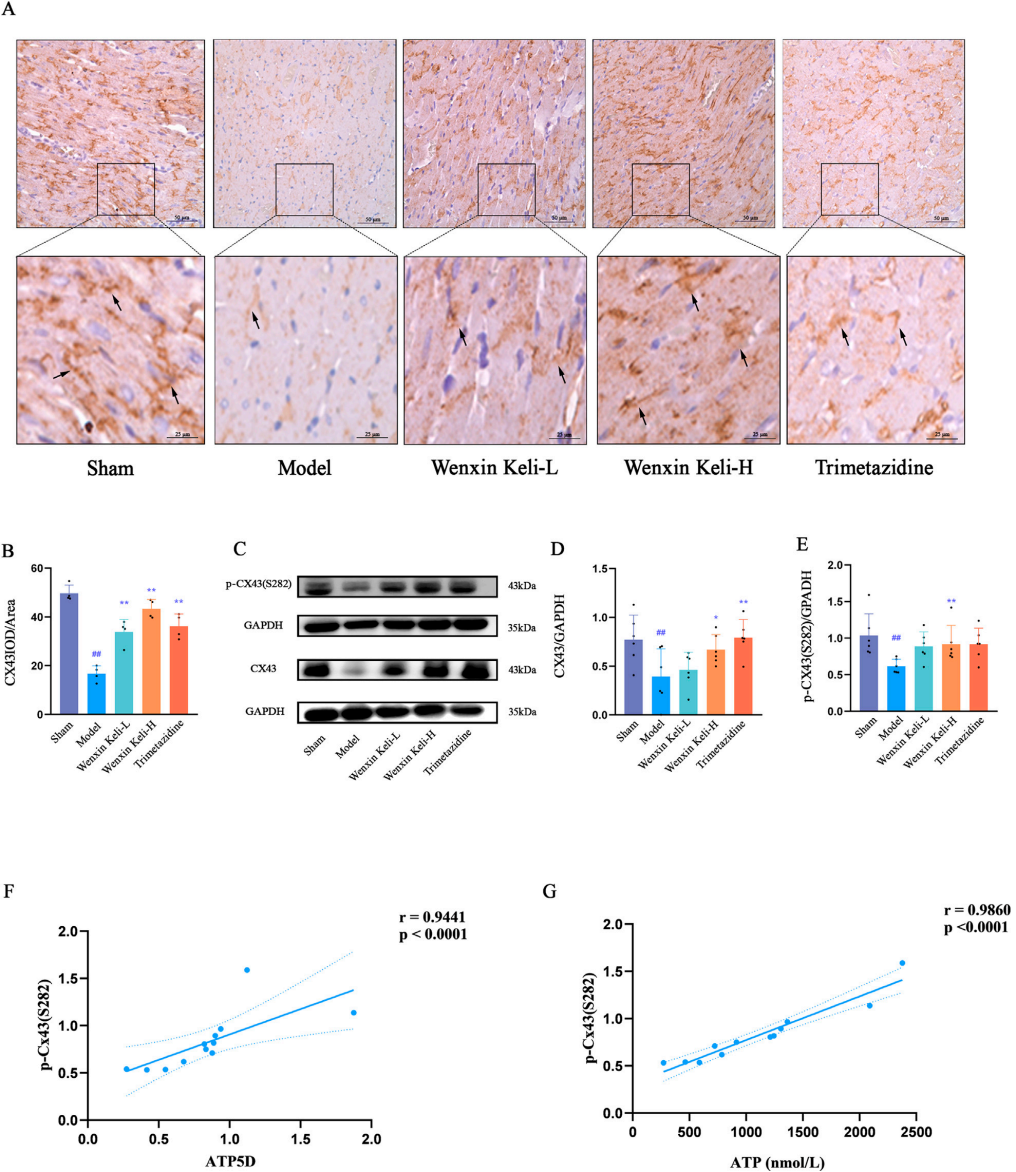
Effects of Wenxin Keli and trimetazidine on Cx43 expression and its correlations with electrophysiological characteristics, ATP, and ATP5D in myocardial infarction-induced heart failure rats **(A)** IHC images of Cx43 expression, **(B)** ImageJ-quantified mean optical density of Cx43 positive expression (n = 4), **(C–E)** WB analyzed Cx43/p-Cx43(S282) levels, quantified via ImageJ (n = 6), **(F,G)** Correlation analysis of ATP5D, ATP, and p-Cx43(S282) data.

WB and immunohistochemistry (IHC) were used to detect Cx43 and p-Cx43(S282) expression (Figures 6A,B). Linear correlation analysis of p-Cx43(S282) with ventricular fibrillation threshold and energy metabolism indices. The WB results showed that compared with the sham operation group, the expression of CX43 and p-CX43 decreased in the model group (Figures 6C–E). IHC revealed organized linear Cx43 localization at cardiomyocyte intercalated discs in the sham group. On the other hand, the model group displayed decreased levels of Cx43, characterized by a dotted distribution pattern and a marked decline in mean optical density. In contrast, results from WB after drug treatments revealed an upsurge in p-Cx43(S282) and Cx43 levels, which was paralleled by an increase in the mean optical density of Cx43 noted in IHC. The correlation coefficients obtained for the expressions of p-Cx43(S282) with ATP and ATP5D were r = 0.9860 and r = 0.9441, respectively, implicating a potential link between the phosphorylation status of Cx43 and energy metabolism (Figures 6F,G).

## 4 Discussion

Cardiovascular disease is a global health issue, with heart failure being its ultimate outcome (Baman and Ahmad, 2020). Extensive research has demonstrated that pathological cardiac changes involve a remodeling of cardiac energy metabolism, where insufficient myocardial energy production exacerbates the complex progression of heart failure. Relevant studies have indicated that regulating energy substrate metabolism could be a potential target for pharmacological interventions to improve the failing heart’s function (Li et al., 2023). Gap junctions, critical for electrical and chemical communication between cardiomyocytes, are implicated in arrhythmias. Cx43, the dominant connexin in cardiac gap junctions, requires ATP-dependent phosphorylation for proper coupling. Heart failure reduces ATP availability, impairing Cx43 phosphorylation and disrupting its coupling function. Traditional Chinese medicine (TCM), such as Wenxin Keli, demonstrates multi-target effects and has been shown to enhance Cx43 expression in heart failure models. However, the mechanisms regulating post-heart failure energy metabolism remain unclear.

The heart’s energy metabolism involves three key processes—substrate utilization, oxidative phosphorylation, and ATP transfer/usage (Bertero and Maack, 2018; Ritterhoff and Tian, 2023), regulated by complex molecular interactions. A central regulator is PGC-1α (Rowe et al., 2010; Riehle and Abel, 2012; Aggarwal et al., 2022), which partners with nuclear receptor coactivators like PPARs and ERRs to govern mitochondrial oxidative metabolism (Panes et al., 2020). Prior work shows its upstream regulators, AMPK and SIRT1, sequentially phosphorylate and regulate PGC-1α (Scarpulla et al., 2012; Luen Tang, 2016), with AMPK enhancing SIRT1 via NAD^+/^NADH ratio modulation (Cantó et al., 2009). These modifications boost PGC-1α activity and its interactions with PPARs/ERRs, amplifying regulation of downstream mitochondrial genes (Fan and Evans, 2015). The SIRT1-AMPK-PGC-1α interplay thus forms a sophisticated regulatory axis for energy homeostasis (Luen Tang, 2016; Cantó and Auwerx, 2009).

After heart failure, the heart shifts energy substrate preference from mitochondrial oxidative metabolism to glycolysis, with reduced PGC-1α expression weakening its upstream/downstream interactions and impairing mitochondrial function (Meng et al., 2021). This study showed Wenxin Keli regulates AMPK, SIRT1, and PGC-1α in myocardial infarction-induced heart failure rats, restoring mitochondrial oxidative phosphorylation by dual modulation of fatty acid/glucose pathways, distinct from trimetazidine, which primarily suppresses fatty acid metabolism (Aggarwal et al., 2022; Riehle and Abel, 2012).

As a central co-regulatory factor, PGC-1α can bind to and activate the transcription of related mitochondrial target gene proteins, and PPARs and ERRs are the crucial transcription factors among them (Giguère, 2023; Fan and Evans, 2015; Oka et al., 2011). In normal adult myocardium, energy supply primarily relies on fatty acid oxidation, which weakens in heart failure. PGC-1α partners with PPARα/δ to activate genes for fatty acid uptake and β-oxidation, promoting expression of proteins like CD36 mediating free fatty acid entry into cardiomyocytes/mitochondria for β-oxidation and CPT-1α (Vannuchi and Pisani, 2025; Cantó and Auwerx, 2009). However, there are slight differences between PPARα and PPARδ in regulating fatty acid oxidation. Both can promote fatty acid metabolism, but PPARα will reduce glucose intake and lower glycolysis levels, which may lead to lipid accumulation and myocardial hypertrophy. Conversely, PPARδ can enhance glucose utilization while facilitating fatty acid metabolism, and this regulatory influence does not lead to lipid buildup or impairment of cardiac function (Fan and Evans, 2015). In relevant studies, PPARδ can significantly increase the expression level of GLUT4 in rats. In the rat model of heart failure after myocardial infarction, the expressions of the above proteins that regulate fatty acids for β-oxidation are all downregulated in cardiomyocytes (Montaigne et al., 2021; Cheng et al., 2018). In this study, Wenxin Keli can not only increase the expressions of PPARα and PPARδ in rats with heart failure after myocardial infarction but also promote the expressions of CPT-1α, CD36, and GLUT4, indicating that Wenxin Keli can regulate fatty acid metabolism and improve the utilization rate of glucose in the mechanism of treating heart failure.

ERRs, including ERRα and ERRγ, serve as distinct PGC-1α co-activators critical for mitochondrial oxidative phosphorylation (Fan and Evans, 2015). These receptors bind to promoters of mitochondrial genes encoding electron transport chain (ETC.) complex subunits and ATP synthase, regulating their expression (Nakadai et al., 2023; Misra et al., 2017; Fan and Evans, 2015). ERRs regulate oxidative phosphorylation proteins, influencing efficiency; ERRα/γ knockout mice show heart failure (Vannuchi and Pisani, 2025; Fan and Evans, 2015). In post-infarction heart failure rats, ERRα/γ, ATP5D expression, and mitochondrial complexes I, II, IV, and V activities decreased; Wenxin Keli improved these parameters, enhancing oxidative phosphorylation. ERRs also work with AMPK to balance the tricarboxylic acid cycle and glycolysis. (Kim and Dyck, 2015). AMPK acts as a cellular energy sensor: activated by low ATP/elevated AMP/ATP ratio, it enhances ERRs activity (Li et al., 2019; Kim and Dyck, 2015). Promoting PDH expression while inhibiting PDK. This drives pyruvate into mitochondria for the tricarboxylic acid cycle, generating acetyl-CoA that inhibits PFK, a glycolytic rate-limiting enzyme. (Nakadai et al., 2023). ERRs’ OXPHOS regulation varies across tissues; in pathological states like cardiac ischemia-reperfusion injury, their role in balancing cardiomyocyte glycolysis amid metabolic dysfunction remains unclear (Nakadai et al., 2023; Lasheras et al., 2021). Model rats exhibited reduced PDH expression with increased PFK/PDK levels, which were reversed by Wenxin Keli, which increased PDH and decreased PFK/PDK, suggesting the drug promotes pyruvate entry into mitochondria for the TAC cycle to enhance energy production.

In heart failure, energy substrate utilization is restricted with reduced uptake/oxidation; inconsistent findings on glucose/fatty acid changes arise from physiological complexity and incomplete research. (Da Dalt et al., 2023; Ritterhoff and Tian, 2023). Advanced heart failure further reduces fatty acid oxidation and glucose utilization, challenging the energy-demanding myocardium, which relies on the creatine kinase system to convert chemical energy to mechanical energy for pumping (Bertero and Maack, 2018). Sustained energy is critical for the myocardium, supporting pumping, gene expression, and protein phosphorylation, which are essential for gap junction proteins like Cx43, which require ATP as a phosphate donor for phosphorylation. (Leybaert et al., 2023; Rodriguez-Sinovas, 2009). In heart failure, inadequate ATP impairs Cx43, a gap junction protein critical for intercellular electrical/metabolite coupling and synchronized contraction (Leybaert et al., 2023; Rodriguez-Sinovas, 2009). The S282 phosphorylation site on Cx43 regulates myocardial survival (inhibiting apoptosis via mitochondrial pathway when phosphorylated, activating pro-death signaling when dephosphorylated) and electrical conduction; S282-mutant mice exhibit arrhythmias and increased apoptosis, highlighting its homeostatic role (Yang et al., 2019) S282-mutant mice show severe ventricular arrhythmias and increased myocardial apoptosis, highlighting S282 phosphorylation’s critical role in cardiomyocyte homeostasis. Wenxin Keli boosts energy production, restoring Cx43 phosphorylation and improving intercellular coupling. Mechanistically, S282 phosphorylation regulates cardiomyocyte survival, electrical conduction, and fibrosis by adjusting its own and adjacent S279 site phosphorylation. (Wang et al., 2025; Huang et al., 2023; Wu et al., 2023; Fu et al., 2022). The drug reverses heart failure metabolic dysfunction by promoting fatty acid/glucose utilization, inhibiting inefficient glycolysis, and restoring mitochondrial oxidation, addressing energy deficits and Cx43-dependent electrical-mechanical synchronization.

**FIGURE 7:**
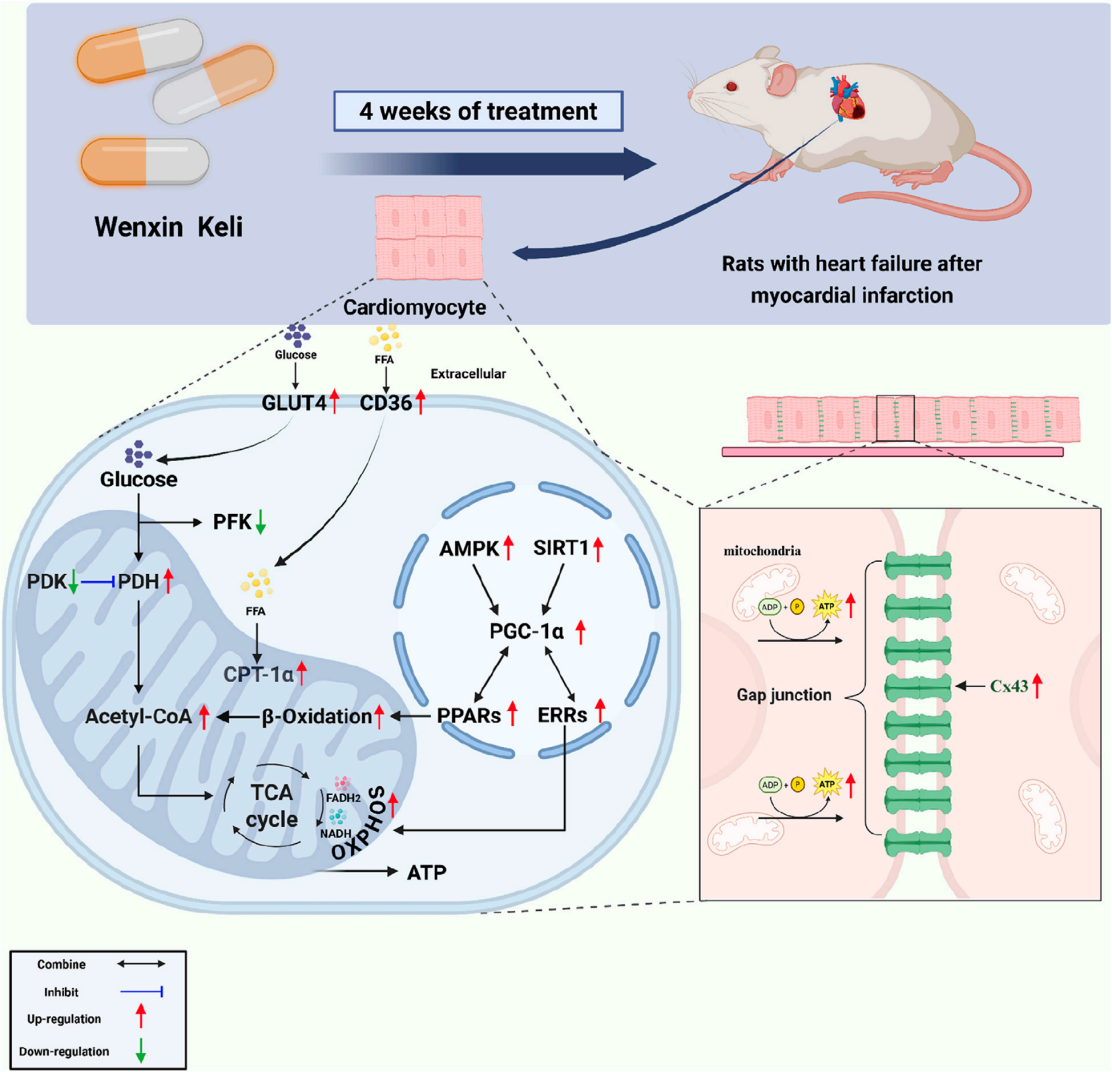
Wenxin Keli regulates energy metabolism and improves Cx43 via the AMPK/SIRT1/PGC-1α pathway.

In summary, Wenxin Keli activates the AMPK/SIRT1/PGC-1α pathway, enhancing PGC-1α interactions with PPARs/ERRs to improve fatty acid/glucose metabolism, restore mitochondrial oxidative phosphorylation, and boost ATP synthesis via the TCA cycle. It also increases AMPK phosphorylation, promoting SIRT1-mediated PGC-1α for positive feedback in pathway activation. As a phosphorylation-dependent gap junction protein, Cx43 benefits from Wenxin Keli-improved ATP availability, enhancing intercellular coupling critical for myocardial electrical and mechanical synchronization (Figure 7). This study conducted an exploratory investigation into the new mechanism of Wenxin Keli, it provided a detailed analysis of its regulation of the AMPK/SIRT1/PGC-1α pathway, promoting the flexible utilization of energy substrates in the myocardium and enhancing the oxidative capacity of mitochondria, thereby delaying the deterioration of cardiac function in heart failure after myocardial infarction and reducing the risk of concurrent ventricular arrhythmias.

## 5 Conclusion

Wenxin Keli enhances cardiac energy metabolism through increased substrate utilization and ATP production mediated by the AMPK/SIRT1/PGC-1α signaling pathway. At the same time, it raises the phosphorylation levels of gap junction proteins, which helps safeguard the Cx43 signaling mechanism, providing a protective effect on the hearts of rats suffering from post-infarction heart failure. However, this study has limitations, such as lacking gene knockout/overexpression experiments for direct validation.

## Data Availability

The raw data supporting the conclusions of this article will be made available by the authors, without undue reservation.
